# RPS9M, a Mitochondrial Ribosomal Protein, Is Essential for Central Cell Maturation and Endosperm Development in *Arabidopsis*

**DOI:** 10.3389/fpls.2017.02171

**Published:** 2017-12-22

**Authors:** Changqing Lu, Feng Yu, Lianfu Tian, Xiaoying Huang, Hong Tan, Zijing Xie, Xiaohua Hao, Dongping Li, Sheng Luan, Liangbi Chen

**Affiliations:** ^1^Hunan Province Key Laboratory of Crop Sterile Germplasm Resource Innovation and Application, Hunan Normal University, Changsha, China; ^2^Department of Plant and Microbial Biology, University of California, Berkeley, Berkeley, CA, United States

**Keywords:** *Arabidopsis thaliana*, central cell, embryo, endosperm, female gametophyte, mitochondrion, ribosomal protein

## Abstract

During double fertilization of angiosperms, the central cell of the female gametophyte fuses with a sperm cell to produce the endosperm, a storage tissue that nourishes the developing embryo within the seed. Although many genetic mutants defective in female gametophytic functions have been characterized, the molecular mechanisms controlling the specification and differentiation of the central cell are still not fully understood. Here, we report a mitochondrial ribosomal protein, RPS9M, is required for central cell maturation. RPS9M was highly expressed in the male and female gametophytes before and after double fertilization. The female gametophytes were defective in the *rps9m* mutant specifically concerning maturation of central cells. The morphological defects include unfused polar nuclei and smaller central vacuole in central cells. In addition, embryo initiation and early endosperm development were also severely affected in *rps9m* female gametophytes even after fertilized with wild type pollens. The RPS9M can interact with ANK6, an ankyrin-repeat protein in mitochondria previously reported to be required for fertilization. The expression pattern and mutant phenotype of *RPS9M* are similar to those of *ANK6* as well, suggesting that RPS9M may work together with ANK6 in controlling female gametophyte development, possibly by regulating the expression of some mitochondrial proteins.

## Introduction

In angiosperms, the egg and sperm cells form within the female and male gametophytes through a series of meiosis, mitosis, and cell fate determination, respectively (Ma and Sundaresan, 2010). During female gametophyte development in most flowering plants, the megaspore mother cell (MMC) undergoes meiosis to produce four haploid megaspores. Three of the megaspores undergo programmed cell death, leaving one survived megaspore to become functional megaspore (FM). Subsequently, the FM cell undergoes three rounds of mitosis without cellularization to produce an eight-nucleate structure. Then migration of nuclei and cellularizations result in a seven-celled female gametophyte consisting of one egg cell, two synergid cells, three antipodal cells, and one central cell containing two polar nuclei (Yang et al., 2010). In *Arabidopsis thaliana* and many other species, two polar nuclei fuse to form a diploid nucleus in the central cell and the central cell vacuole enlarged before fertilization. (Yadegari and Drews, 2004).

By genetic and molecular approaches, a number of genes have been identified to be involved in female gametophyte development (Yadegari and Drews, 2004; Kaegi and Gross-Hardt, 2007; Liu and Qu, 2008; Sundaresan and Alandete-Saez, 2010; Yang et al., 2010; Martin et al., 2014), and some of them display maternal effects on seed formation (Huh et al., 2008; Berger and Chaudhury, 2009; North et al., 2010). In the past decades or so, mutants defective in central cell development have also been described, including *FEM111*/*AGL80* (Portereiko et al., 2006a), *AGL61* (Bemer et al., 2008; Steffen et al., 2008), and the *BiP* genes (*BiP1* and *BiP2*) (Maruyama et al., 2010). *AGL61* and *AGL80* encode the type I MADS-domain transcription factors and mutation of either of them results in smaller central cell with reduced vacuole. Consequently the central cell in either *agl61* or *agl80* mutant fail to give rise to endosperms after fertilization. Yeast two-hybrid (Y2H) assay show that AGL61 interact with AGL80, suggesting that these two transcription factors may function as a heterodimer in controlling the expression of downstream genes during central cell development. The *BiP* genes encode molecular chaperones of the Hsp70 family in the endoplasmic reticulum. The central cell in *bip1bip2* double mutant show defects in the fusion of polar nuclei, which may result from altered nuclear membrane. After fertilization, the mutant shows aberration in endosperm nuclear division, indicating that central cell maturation plays an important role in endosperm development.

Mitochondria also play a critical role in female gametophyte maturation. Mutations in some genes encoding mitochondrial proteins cause defects in female gametophyte. These include *RPL21M/NFD1* (Portereiko et al., 2006b), *FIONA/SYCO* (Kaegi et al., 2010), *GCD1* (Wu et al., 2012) and *MSD1/OIWA* (Martin et al., 2013). *RPL21M* encodes the mitochondrial 50S ribosomal subunit L21 and is essential for the fusion of polar nuclei during female gametophyte maturation and the male-female gametophyte recognition during double fertilization. *FIONA* encodes the cysteinyl t-RNA synthetase and requires for the central cell development and mitochondrial cristae integrity. *GCD1* encodes the ribosomal protein L20 which is enriched in gametophytes and plays important roles in fusion of polar nuclei, maturation of female gametes, embryogenesis initiation, and endosperm development. *MSD1* encodes a mitochondrial Mn-superoxide dismutase, which is critical for distribution of reactive oxygen species (ROS) during embryo sac patterning. All these genes described above are important for central cell maturation and endosperm development, indicating the importance of mitochondria in female gametophyte maturation.

Our previous work identified *ANK6* gene as required for male–female gamete recognition. ANK6 is an ankyrin protein located in the mitochondria and likely functions as a scaffold to mediate protein–protein interactions through its ankyrin repeat domains. Indeed, we found a member of σ-transcription initiation factor, SIG5, as a partner for ANK6. Like in *ank6* mutant, a proportion of *sig5* embryo sacs are also arrested at late stages of female gametophyte development (Yu et al., 2010). In the same yeast two hybrid screen, a mitochondrial ribosomal small subunit protein, designated RPS9M, showed interaction with ANK6. *RPS9M* is highly expressed in both male and female gametophytes just like *ANK6*. The *rps9m* mutants have female gametophyte defects and, in particular, the central cells showed smaller vacuoles and defect in the fusion of polar nuclei. Although *rps9m* embryo sacs are capable of double fertilization, embryogenesis initiation and endosperm proliferation are both defective after fertilization whether mutant or wild type pollens are used for pollination. Together, these results suggest that RPS9M functions with ANK6 and possibly other proteins (e.g., SIG5) in the control of mitochondrial gene expression during female gametophyte.

## Materials and Methods

### Plant Materials and Growth Conditions

*Arabidopsis thaliana* (ecotype Columbia-0) plants were used in this study unless otherwise indicated. Seeds were surface sterilized in 70% ethanol for 5 min, stratified at 4°C for 2 days, and grown on half-strength Murashige and Skoog (Sigma ^1^) medium at 22°C under 16:8 h light: dark cycles for a week. Then plants were transferred to soil and incubated in a constant-temperature room at 22°C under 16 h light/8 h dark cycles. *Agrobacterium*-mediated transformation were as described previously (Clough and Bent, 1998). The transgenic lines were selected on 0.5 × Murashige and Skoog medium (Sigma) containing 50 μg/mL kanamycin (for pBI101-based vectors) or 30 μg/mL hygromycin B (for pCAMBIA 1300-based vectors).

### Plasmid Constructs

The *RPS9M* cDNA is amplified by RT-PCR using the primer pair *RPS9M-F* and *RPS9M-R* and ligated into the pMD18-T vector (TaKaRa). To make the *ProRPS9M*-GUS construct for histochemical analysis, a 2.2-kb fragment upstream of the ATG starting codon of *RPS9M* was PCR amplified using the primers *RPS9M*-PF and *RPS9M*-PR and cloned into the pMD18-T vector, then subcloned into the pBI101.2 vector using the same primers. For subcellular localization analysis, the *RPS9M* CDS was amplified without its stop codon using the primer pair *RPS9M-*GF and *RPS9M-*GR, subsequently digested with *Xba* I and *Bam*H I, respectively, and then inserted into the plant binary vector pMD1-GFP to make the 35S-*RPS9M-GFP* fusion construct.

For CRISPR/Cas9 plant expression vectors construction. The chimeric sgRNA for *RPS9M* was constructed by cloning annealed oligos RPS9M-sgF and RPS9M-sgR into pBlueScript SK-sgRNA, then both the sgRNA and hSpCas9 were subcloned into the expression vector pCAMBIA1300 (Supplementary Figure S2A) as previously described (Feng et al., 2013; Mao et al., 2013).

### Histochemical GUS Analysis

Transformed *Arabidopsis* lines carrying the *pRPS9M-GUS* fusion were selected based on Kanamycin resistance. The promoter activity was reflected by GUS activity visualized in the plant tissues using 5-bromo-4-chloro-3-indolylb-D-glucuronide (X-Gluc) as a substrate according to published protocols (Jefferson et al., 1987). In details, inflorescences and 2 week old seedlings were incubated in GUS staining solution [10 mg/mL X-Gluc, 0.1 M K_3_(Fe(CN)_6_), 0.1 M K_4_Fe(CN)_6_⋅3H_2_O, 1 M Na_2_EDTA, 0.2 M Na_3_PO_4_ (pH7.0)] at 37°C overnight. Then the plant tissues were decolorized for 30 min in each of the gradient ethanol solution (30, 40, 50, 60, 70%) and incubated in 75% ethanol at 37°C overnight. The samples were examined and photographed with an Olympus SZX12 microscope equipped with a camera.

### Yeast Two-Hybrid (Y2H) Screen and Analysis

We used the CLONTECH Matchmaker GAL4 Two-Hybrid System 3 for the yeast (*Saccharomyces cerevisiae*) two-hybrid screen and analysis. For performing Y2H screen of *Arabidopsis* cDNA library, according to the method described as previous (Yu et al., 2010). The *ANK6* CDS were fused to GAL4 DNA-binding domain in pGBKT7. AH109 cells were first transformed with pBD-ANK6 plasmid, then the transformants were transformed with the *Arabidopsis* cDNA library cloned in the prey vector pACT. The transformed cells were plated on synthetic dropout (SD) selection medium that lacked Trp, Leu, and His supplemented with 20 mM 3-AT to reduce the growth of false-positive colonies. The plates were incubated at 30°C for 3–12 days. The prey plasmid DNAs were isolated from yeast, transformed and isolated from *Escherichia coli*, and retransformed into yeast cells containing either the empty vector or the vector with bait to verify growth further. The clones that continued to grow in the -His and +3-AT medium after retransformation were selected for DNA sequencing. For Y2H analysis the interactions between RPS9M and ANK6, the *RPS9M* CDS and truncated *RPS9M* variants were fused to the GAL4 activation domain in pGADT7. Yeast strain AH109 was cotransformed with combinations of pGADT7 and pGBKT7 constructs and selected on SD medium lacking Leu and Trp (SD-LW). Cotransformants were then assayed for interaction and activation of the His and adenine reporter genes on SD medium lacking Leu, Trp, His, and adenine (SD-LWHA). For this, fresh colonies were grown in SD-LW at 30°C overnight to an OD_600_ of 1–2, the cells were pelleted and resuspended in 0.5 M sorbitol to an OD_600_ of 0.5, and 5 μL of each cell suspension was spotted on SD-LWHA plates using a multichannel pipette and grown at 30°C for 2–3 days.

### Bimolecular Fluorescence Complementation (BiFC) Assays

The ORF sequence of *ANK6* and *RPS9M* was cloned into the plasmid pE3308 and pE3449 using the primer pairs list in Supplementary Table S2. Protoplasts were isolated from 4 week old *Arabidopsis* rosette leaves. Transient protoplast expression was performed using the polyethylene glycol transformation method as described previously (Sheen, 2001).

### CLSM and Central Cell Vacuole Size Calculation

The confocal observation of ovules was performed according to the method described previously (Christensen et al., 1997) with slight modifications. Inflorescences were harvested and fixed in 4% glutaraldehyde (in 12.5 mM cacodylate, pH 6.9), and a vacuum was applied for the initial 20 min, after which they were in fixative overnight at room temperature. After fixation, the tissues were dehydrated through a conventional ethanol series with 20 min per step. After the dehydration, the tissue was cleared in 2:1 (v/v) benzyl benzoate: benzyl alcohol for a minimum of 1 h. Pistils or siliques were dissected, mounted with immersion oil, and observed with a Zeiss LSM510 META laser scanning microscope (Zeiss, Jena, Germany) with a 488-nm argon laser and an LP 530 filter. Images were edited with Zeiss LSM Image Browser software and Photoshop software. The central cell vacuole size was calculated according to the method as described previously (Wu et al., 2012). Single section image was captured when scanning to the largest sectional view of the central cell vacuole, and the size of central cell vacuoles were calculated using Image J software.

## Results

### RPS9M Interacts with Ankyrin Protein ANK6

**FIGURE 1:**
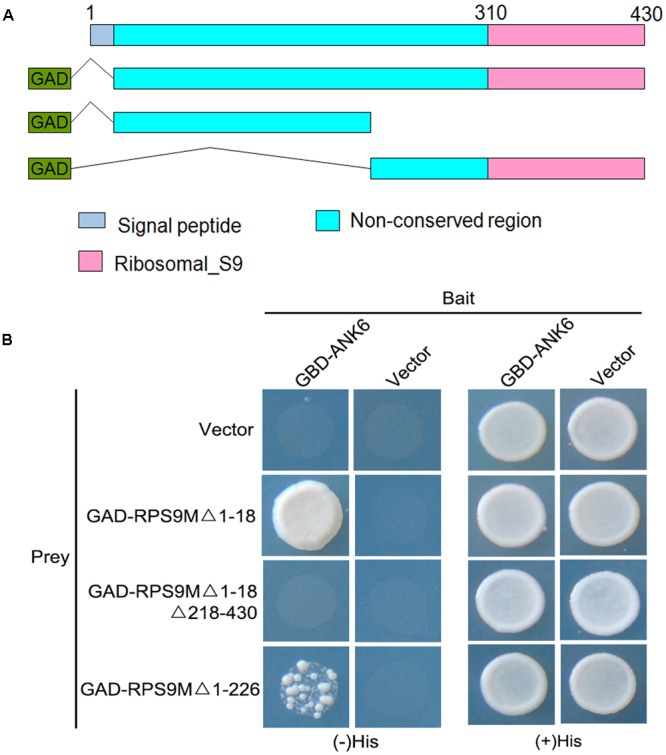
Interaction between RPS9M and ANK6 determined by Y2H analysis. **(A)** Diagram of the full-length and truncated RPS9M constructs with specific deletions. **(B)** Y2H analysis of ANK6 interacting with full-length and truncated RPS9M.

The ankyrin repeats are generally recognized as protein–protein interaction modules. We previously showed that mitochondrial ANK6 protein functions in double fertilization, suggesting that a protein complex mediated by this ankyrin repeat protein might participate in ovule development. We performed a Y2H screening by using ANK6 (amino acid residues 21–174) as a bait to screen a prey cDNA library. Several positive clones were obtained, including *RPS9M*, a bacterial-origin mitochondrial-type ribosomal protein S9 gene (AT3G49080) (Bonen and Calixte, 2006). It encodes a 430-aa protein, with a Ribosomal_S9 domain at the C terminus (amino acid residues 310–430) and a non-conserved region at the N-terminus, according to the SMART protein domain prediction program (**Figure 1A**). The sequence of the Ribosomal_S9 domain shows high similarity to the RPS9 protein of *Escherichia coli* and chloroplast ribosomal protein S9 of *Arabidopsis*. To identify the ANK6-interacting domain, three truncated RPS9M variants were fused with GAL4 activation domain (**Figure 1A**). The interaction between these variants and ANK6 were assayed by using the Y2H system (**Figure 1B**). ANK6 interacts with the C-terminus Ribosomal_S9 domain of RPS9M. RPS9M lacking the non-conserved N-terminus showed a reduced interaction activity with ANK6, suggesting a role for the N-terminus in regulating the interaction of RPS9M with ANK6 (**Figure 1**).

### RPS9M Is a Mitochondrial Localized Protein and Interacts with ANK6 in Mitochondria

**FIGURE 2:**
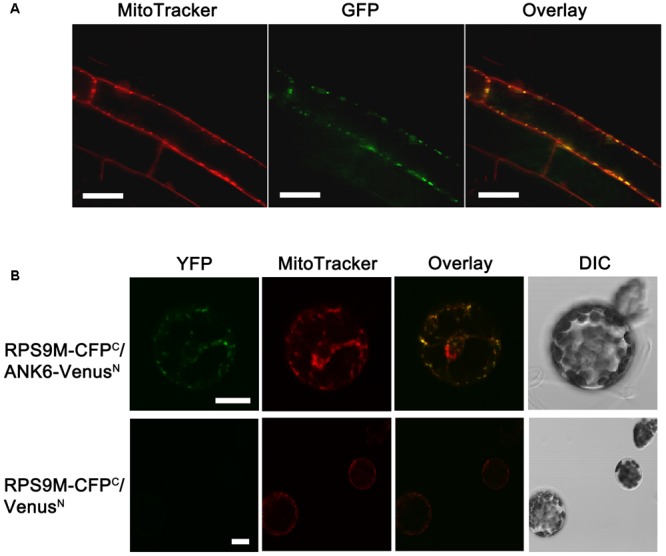
The subcellular localization of RPS9M in plant and interaction between RPS9M and ANK6 determined by BiFC analysis in mesophyll protoplasts. **(A)** Subcellular localization of RPS9M. From left to right, MitoTracker, GFP fluorescence and merged images. **(B)** The interaction between RPS9M and ANK6 determined by BiFC analysis in mesophyll protoplasts. Full-length RPS9M protein was fused to C-terminal CFP (CFP^C^), and full-length ANK6 was fused to N-terminal Venus (Venus^N^). Mesophyll protoplasts were cotransfected with the RPS9M-CFP^C^ and ANK6-Venus^N^ fusion constructs (Upper) or with the RPS9M-CFP^C^ fusion and Venus^N^ construct (Lower). From left to right, YFP signals (green, observed with GFP filter), MitoTracker (red) stained mitochondria, merged images and DIC. Bars = 20 μm.

The RPS9M protein contains a mitochondrial pre-sequence that targets the protein to mitochondria according to SUBAcon. To investigate the subcellular location, an *RPS9M-GFP* gene cassette driven by the cauliflower mosaic virus 35S promoter was constructed and introduced into wild type plants (Supplementary Figure S4). The GFP fluorescent signals were detected in the roots of these transgenic plants under confocal microscopy. MitoTracker dye was used as a control to stain mitochondria of the roots. In the root epidermal cells of a *35S-RPS9M-GFP* line, the green fluorescent signals were colocalized with the MitoTracker fluorescent signals (**Figure 2A**), indicating that RPS9M protein was targeted to mitochondria.

To confirm the interaction between RPS9M and ANK6, we performed bimolecular fluorescence complementation assays in plant cells. We co-expressed RPS9M tagged with C-terminal CFP and ANK6 tagged with N-terminal Venus in mesophyll protoplasts. The yellow/green fluorescence signals indicated interaction between RPS9M and ANK6. Mitochondrial dye was used to localize mitochondria. As shown in **Figure 2B**, the yellow/green fluorescence and the red dye signals were overlapped, supporting the notion that RPS9M and ANK6 interact with each other in mitochondria.

### *RPS9M* Is Expressed in Both Male and Female Gametophyte

**FIGURE 3:**
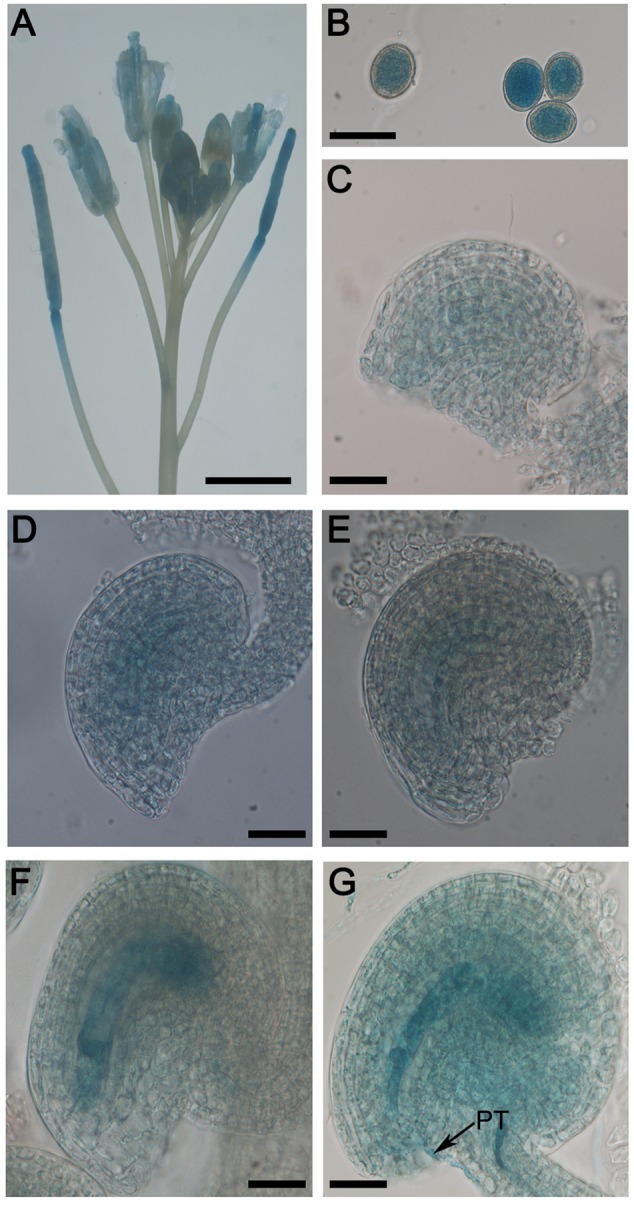
Expression patterns of RPS9M by GUS reporter analysis. Histochemical analysis of *RPS9M* promoter-driven GUS reporter expression in transgenic *Arabidopsis* plants. GUS signals (indicated in blue) were observed in inflorescence **(A)**, pollen grains **(B)** and different developmental stages of female gametophytes **(C–G)**. Inflorescence **(A)**, mature pollen grains **(B)**, FG1 stage **(C)**, FG2-FG3 stage **(D)**, FG4 stage **(E)**, FG5–FG7 stage **(F)**, ovule with penetrating pollen tube (PT, arrow) **(G)**. Bars = 5 mm **(A)** and 20 μm **(B–G)**.

In order to reveal the function of *RPS9M* gene, we first examined the expression pattern of *RPS9M* using transgenic plants harboring the reporter construct with *RPS9M* promoter fused to the β-glucuronidase (GUS) gene. Seven GUS transgenic lines were selected and their GUS activities were identified with similar expression patterns. Strong GUS activities were detected in inflorescence particularly in the anthers and pistils of T_2_ transgenic lines (**Figure 3A** and Supplementary Figures S1A,B). In anthers, GUS activities were detected throughout the developmental stages, especially in the mature pollen grains (**Figure 3B** and Supplementary Figures S1A,B). In pistils, GUS activities were detected in female gametophytes at different developmental stages (**Figures 3C–G**), particularly in the mature female gametophyte stage (**Figure 3F**). The *RPS9M* promoter was also highly active during double fertilization (**Figure 3G**). After fertilization, GUS signals were detected in young siliques, especially in embryo and endosperms (Supplementary Figures S1B,D,E), but no GUS signal was detected in mature seeds (Supplementary Figure S1C). GUS signals were also observed in young leaves and root tips (Supplementary Figure S1F). These expression patterns suggest that RPS9M may participate in the development of male and female gametophytes as well as in embryogenesis.

### *RPS9M* Mutant Displayed Empty Seed Sets in Siliques

**FIGURE 4:**
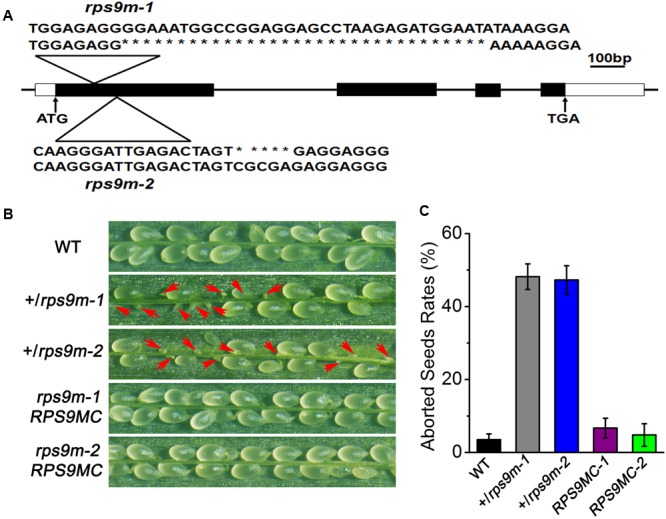
Phenotypic observation of *+/rps9m* siliques. **(A)** Schematic diagram of *RPS9M* gene structure and the mutation position in *rps9m-1* and *rps9m-2* mutant. The ATG start codon and TGA stop codon are shown as indicated. The mutation position and deleted nucleotides was determined to the first exon by PCR and sequencing (triangle above the gene diagram). **(B)** Phenotype of the +/*rps9m* plant. In contrast to wild type (upper), half of seeds in selfed +/*rps9m-1* and +/*rps9m-2* siliques were aborted during development (arrowheads, middle), whereas the complemented transgenic plants of *rps9m-1* and *rps9m-2* (*RPS9MC1* and *RPS9MC2*) recovered the phenotype to the wild type level (lower). **(C)** Frequencies (%) of the aborted seeds in per silique of wild type, +/*rps9m-1*, +/*rps9m-2* and *RPS9MC1* and *RPS9MC2* plants. Error bars represent ± SD.

To determine the function of *RPS9M* gene in plant development, CRISPR/Cas9 gene editing approach was adopted to produce mutations in the *RPS9M* gene (Jiang et al., 2013; Mao et al., 2013; Zhang et al., 2014). We designed two sgRNA that targeted the coding sequence of *RPS9M* gene and transformed into wild type plants. A series of heterozygous mutants were obtained in the T_1_ generation. Most of the mutants involved insertions or deletions of a few nucleotides, with rare cases of sizable deletions of 30 or more nucleotides (Supplementary Figures S2B–D). In this study we chose two mutant lines for further research, one contain a deletion of 32 nucleotides, equivalent to the sequence of +271–+302 (named as *rps9m-1*) and the other contain a deletion of 5 nucleotides equivalent to the sequence of +375–+379 (named as *rps9m-2*) (**Figure 4A**). Genetic segregation in T_2_ generation produced mutant plants that did not contain T-DNA insertion sequences or sgRNA/Cas9 fragments. Genotyping of +/*rps9m-1* and +/*rps9m-2* in T_3_ and T_4_ generation led to identification of several heterozygous mutant plants, but no homozygous mutant plants. No homozygous mutant was obtained even after +/*rps9m* plants were selfed, implying that the *rps9m* mutant allele may cause gametophyte defect or homozygous embryo lethality. The heterozygous plants grew normally in the vegetative stage. However, after fertilization, about 50% ovules in both +/*rps9m-1* and +/*rps9m-2* siliques were aborted at early stage (**Figures 4B,C**). Analysis of the progeny of +/*rps9m-1* and +/*rps9m-2* plants and other mutant lines in T_4_ generation revealed a segregation ratio of wild type to heterozygous plants close to 1:0.2 (Supplementary Table S1). These data suggested that not only the female gametophyte, but also the male gametophyte were defective when *RPS9M* function was disrupted.

To further examine the function of *RPS9M* in reproduction, reciprocal cross-tests between the heterozygous +/*rps9m-1* mutant and wild type plants were performed. When +/*rps9m-1* pistils were pollinated with wild type pollens, only a few heterozygote plants were identified in the progeny, the transmission of *rps9m-1* allele through female gametophyte was reduced to 3% (*n* = 151; *P*< 0.001) (**Table 1**). In addition, siliques resulting from this cross exhibited about 50% aborted seed sets. These observations indicate that *RPS9M* lose-of-function mutation almost completely disrupt the female gametophyte function. When wild type ovules were pollinated with +/*rps9m-1* pollens, we found the transmission efficiency of the *rps9m-1* male gametophyte was significantly reduced (TE = 21%; *n* = 167; *P*< 0.001), compared with that of the wild type allele (**Table 1**), indicating that the male gametophyte function is also affected, albeit to a lesser extent compared to the defect of the female gametophyte. Similar results were obtained when reciprocal cross-tests were done between +/*rps9m-2* and wild type plants that the transmission of female gametophyte was almost completely blocked and the transmission of male gametophyte was reduced to 24% (**Table 1**). These results demonstrate that loss of *RPS9M* function indeed cause defects in both two gametophytes, especially the female gametophyte.

**Table 1:** Transmission of *rps9m* alleles to F1 progenies after reciprocal crosses.

Parental genotypes		Progeny genotypes		Expected ratio	Observed ratio
Female	Male	WT	+/*rps9m*		
+/*rps9m-1*	WT	146	5	1:1	1:0.03^a^
WT	+/*rps9m-1*	138	29	1:1	1:0.21^a^
+/*rps9m-2*	WT	154	3	1:1	1:0.02^a^
WT	+/*rps9m-2*	187	44	1:1	1:0.24^a^

*^a^Significantly different from the expected 1:1 segregation ratio based on χ^*2*^ values (*P*< 0.001); +, *RPS9M* allele.*

To verify that the observed phenotypes were caused by loss-of-function of the *AtRPS9M* gene, we performed genetic complementation experiment by introducing *35S-RPS9M-GFP* construct into +/*rps9m-1* and +/*rps9m-2* mutant plant. In the T_2_ populations, we obtained 5 independent lines that were homozygous for both the *rps9m-1* allele and the *RPS9M* transgene, and 3 independent homozygous lines for *rps9m-2* and *RPS9M* transgene. In the progenies of these transgenic lines, silique seed sets were fully restored to the wild type level (**Figures 4B,C**), demonstrating that the *RPS9M* transgene fully complemented the *rps9m* phenotype.

### Female Gametophyte Development Is Affected in *rps9m* Mutants

**FIGURE 5:**
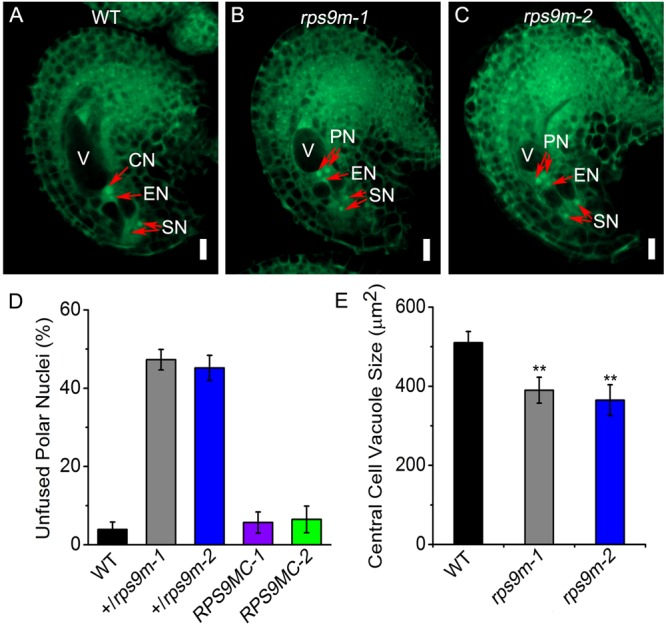
The observation of central cell development in *rps9m* female gametophyte by CLSM. **(A)** Wild type female gametophyte at FG7 stage. The female gametophyte contains two synergid nucleus, one egg nucleus and a secondary central cell nucleus. **(B)** The *rps9m-1* female gametophyte at the same pistil. The two polar nucleus still unfused and the central cell vacuole is smaller compared to that in wild type. **(C)** The *rps9m-2* female gametophyte at the same stage. **(D)** Frequencies (%) of unfused polar nuclei in wild type, +/*rps9m-1*, +/*rps9m-2* and the complemented transgenic plants of +/*rps9m-1* and +/*rps9m-2* at this stage. **(E)** The central cell vacuole size of wild type, *rps9m-1* and *rps9m-2*. CN, central cell nucleus; EN, egg cell nucleus; PN, polar nucleus; SN, synergid nucleus; V, vacuole. Error bars represent ± SD. Bars = 10 μm. ^∗∗^*P*< 0.05, Student’s *t*-test.

Genetic analysis of the *rps9m* mutant suggested a defect in female gametophyte function. To further examine this defect, we monitored ovule development in wild type and +/*rps9m* plants by using whole-mount clearing and confocal laser scanning microscopy (CLSM). We first analyzed female gametophytes at the terminal development stage (stage FG7). We emasculated the wild type, +/*rps9m-1* and +/*rps9m-2* flowers (stage 12c) and kept them growing for 24 h, then fixed the ovule tissues for confocal analysis. Wild type female gametophytes at this stage have one egg cell, one central cell, and two synergid cells, whereas the three antipodal cells were undetected. The two polar nuclei have fused to form a diploid nucleus in most of the central cells (**Figure 5A**).

On the other hand, ovules in +/*rps9m-1* and +/*rps9m-2* heterozygous flowers at the same stage showed differences from the wild type. Specifically, about 47% (*n* = 720) ovules in +/*rps9m-1* plants and 45% (*n* = 630) embryo sacs in +/*rps9m-2* pistils contained unfused polar nuclei (**Figures 5B–D**), indicating defections of polar nuclei fusion in *rps9m* female gametophytes. In addition, we measured the central cell vacuole size in the embryo sacs, as shown in **Figure 5E**, the wild type central cell vacuole size at this stage usually developed to about 510 μm^2^, but in *rps9m* sacs, the size of central cell vacuole was only about 390 μm^2^ in *rps9m-1* and 365 μm^2^ in *rps9m-2* which were significantly smaller than those of wild type. We next observed the morphology of egg cells and synergid cells, no obvious difference was found among *rps9m-1, rps9m-2* and wild type (**Figures 5A–C** and Supplementary Table S4). These observations suggesting that disruption of *RPS9M* inhibited fusion of polar nuclei and affected central cell maturation. To further exclude the possibility that *RPS9M* affects the earlier developmental stages of female gametophyte, we examined female gametophytes from flowers at earlier development stages. No phenotypic variations appeared between wild type and *rps9m* ovules during nuclear mitosis stages (Supplementary Figure S3 and Supplementary Table S3), indicating that macrospore nuclear proliferations and migrations were not affected in *rps9m* female gametophytes.

Together, the data above indicate that the mutation of *RPS9M* specifically affects the final maturation process of the central cell during female gametophyte development.

### Pollen Tube Guidance and Fertilization Is Not Impaired in *rps9m*

**FIGURE 6:**
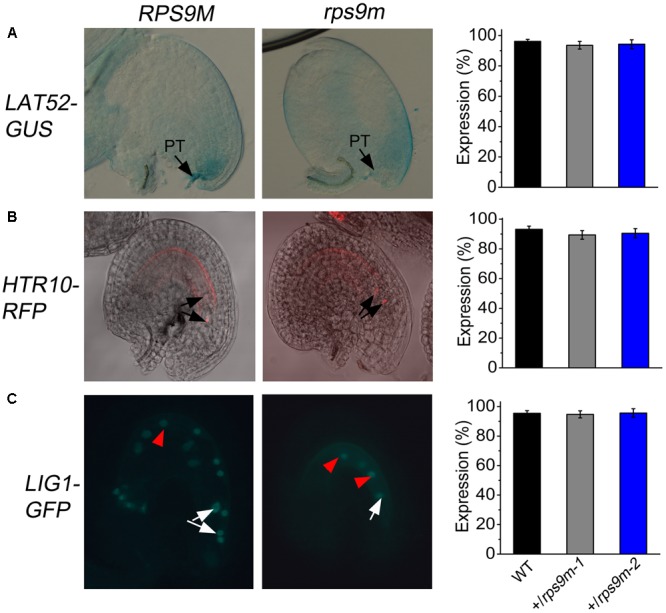
The observation of the pollen tube guidance of *rps9m* female gametophytes. **(A)** The attraction of *rps9m* female gametophyte to pollen tube determined by GUS reporter. Wild type and +/*rps9m* pistils were pollinated with *LAT52-GUS* pollens. Pollen tube entry into the female gametophytes was visualized by GUS staining at 1 DAP (black arrowheads). The percentages of GUS positive ovules per pistil in wild type and +/*rps9m* are shown. **(B)** The attraction of *rps9m* female gametophyte to pollen tube determined by RFP reporter. The pistils of wild type and +/*rps9m* plant were pollinated with pollens expressing sperm-specific marker *HTR10-mRFP*. Ovules were dissected from the pistils and observed by CLSM at 8–10 h after pollination. The arrowheads show two fertilized sperm nuclei. And the percentages of RFP positive ovules per pistils in wild type and +*/rps9m* were shown. **(C)** The fertilization of *rps9m* female gametophyte to sperms determined by GFP reporter. The pistils of wild type and +/*rps9m* plant were pollinated with *LIG1-GFP* pollens. Ovules were dissected from the pistils and observed at 24 h after pollination. The fertilized embryo or zygote are shown (white arrowheads) and endosperm nuclei are visible (red arrowheads). PT, pollen tube. Error bars represent ± SD. Bars = 20 μm.

Several studies show that central cell defects sometimes affect other processes, such as pollen tube guidance and fertilization (Christensen et al., 2002; Portereiko et al., 2006b; Chen et al., 2007). In order to test whether *rps9m* embryo sacs affect pollen tube guidance, pollens from a transgenic line expressing *pLAT52-GUS* were used as donors to pollinate wild type, +/*rps9m-1* and +/*rps9m-2* pistils (Twell et al., 1989). The ovules were collected and GUS activities were analyzed at 24 h after pollination. Almost all ovules in wild type (96%, *n* = 190), +/*rps9m-1* (93%, *n* = 240) and +/*rps9m-2* (94%, *n* = 220) pistils had a pollen tube entered (**Figure 6A**), indicating that pollen tube guidance was not impaired in *rps9m* female gametophyte. To further investigate whether mutation of *RPS9M* in female gametophyte impact fertilization process, a transgenic line expressing *pHTR10-mRFP1*, a sperm-nuclear protein labeling monomeric red fluorescent protein, was used as pollen donors (Ingouff et al., 2007). We pollinated wild type, +/*rps9m-1* and +/*rps9m-2* pistils with *pHTR10-RFP1* pollens. By 12 h after pollination, the RFP signals can be detected in 93% (*n* = 96) ovules of wild type, 89% (*n* = 117) ovules of +/*rps9m-1* and 90% (*n* = 120) ovules of +/*rps9m-2* plants at positions of the egg cell and central cell (**Figure 6B**), indicating disruption of *RPS9M* in female gametophyte does not impact pollen tube reception and sperm cell migration. The possibility of fertilization was evaluated by duration of RFP signals in *rps9m* female gametophyte after pollination, since the RFP signals elapsed quickly as soon as the fertilization was completed. About 1–2 days after pollination, no RFP signals can be detected in both wild type and *rps9m* female gametophytes, suggesting the double fertilization process is completed in not only wild type but *rps9m* female gametophytes as well. To ascertain that the double fertilization occurred in *rps9m* female gametophyte, a transgenic line expressing DNA LIGASE 1 nuclear protein and green fluorescent protein (GFP) fusion protein is used as paternal line (Ingouff et al., 2009). We therefore pollinated *+/rps9m-1*, +/*rps9m-2* and wild type pistils with pollens containing LIG1-GFP. Up to 1 days after pollination (DAP), the paternal expression of LIG1-GFP labeled both the embryo and endosperm in seeds produced from WT (95%, *n* = 135), +/*rps9m-1* (94%, *n* = 170) and +/*rps9m-2* (96%, *n* = 174) pistils (**Figure 6C**), indicating double fertilization is not affected in *rps9m* female gametophyte.

### Embryogenesis and Endosperm Proliferation Are Defective in *rps9m* Mutants

**FIGURE 7:**
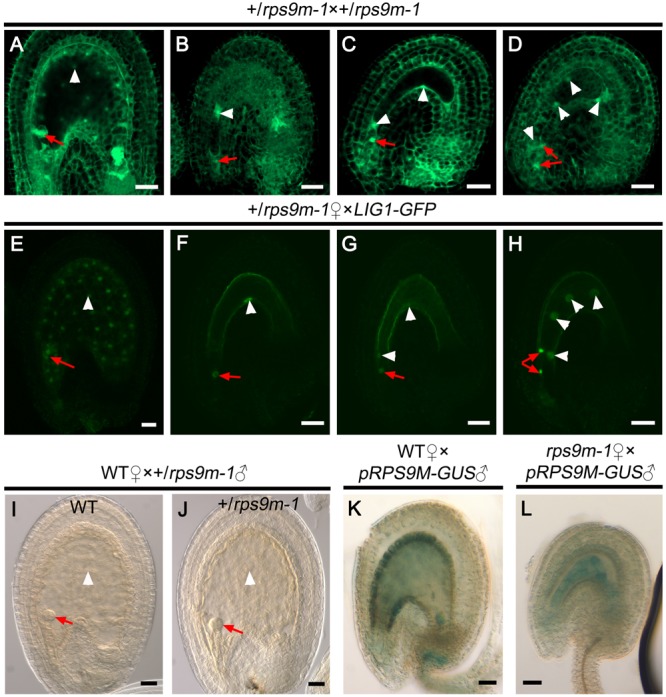
The zygote and early endosperm development in *rps9m* ovules after fertilization. **(A–D)** Observation of embryo and endosperm development in +/*rps9m-1* siliques after fertilization. Pistils of +/*rps9m-1* plant were pollinated with +/*rps9m-1* pollens, and the seeds were observed at 2 DAP by CLSM. **(A)** Wild type seed showed normal embryo development and endosperm proliferation. **(B)** The seed arrested immediately after fertilization in +/*rps9m-1* silique. **(C)** The seed arrested at zygotic embryo stage contain two endosperms in +/*rps9m-1* silique. **(D)** The seed arrested at two-celled proembryo stage with four endosperms from +/*rps9m-1* siliques. **(E–H)** Pistils of +/*rps9m-1* plant were pollinated with wild type pollen expressing nucleus-specific marker *LIG1-GFP*. Ovules were dissected from the pistils and observed by CLSM at 2 DAP. GFP signals can be detected in all fertilized ovules. The *RPS9M* ovules developed normally **(E)**, but *rps9m-1* ovules arrested at different stages **(F–H)**. **(I,J)** Seeds development after wild type female gametophytes were fertilized with +/*rps9m-1* pollens. Both embryo and endosperms developed normally in wild type **(I)** and +/*rps9m-1* **(J)** seeds. **(K,L)** GUS staining of paternal expression of *pRPS9M-GUS* in the developing seeds. The wild type **(K)** or *rps9m-1* **(L)** female gametophyte were pollinated with *pRPS9M-GUS* pollens, and observed at 1 DAP. The red arrows indicate the embryos; the white arrowheads indicate the endosperm nuclei. Bars = 20 μm.

In wild type ovules, fertilized egg cell (zygote) undergoes mitosis to form the embryo and the fertilized central cell gives rise to endosperm following fertilization, ultimately developing into mature seeds. But in *+/rps9m-1* siliques approximately 50% seeds aborted, the observed seed abortion phenotype was probably due to abnormal development of the embryo and/or the endosperm (Yadegari and Drews, 2004; Berger et al., 2006). We therefore analyzed embryo and endosperm development in *+/rps9m-1* siliques at different time points after pollination. Briefly, the *+/rps9m-1* flowers were self-pollinated. By 2 days after pollination (DAP), 91.2% of the young seeds (*n* = 437) contained both an embryo and endosperms. However, 41.5% of the seeds were quite different from the other half in size and endosperm cavity (**Table 2**). The wild type endosperm usually had 16 or more endosperm nuclei. In contrast, majority of the mutant endosperms only had two (19.5%) or four (14.6%) nuclei, and about 7.4% mutant endosperm arrested immediately. The mutant embryo development was also arrested, about 22% of the embryos arrested at the zygote stage, and 19.6% of them divided once to produce a 2-cell embryo before arrested in +/*rps9m-1* silique (**Figures 7A–D** and **Table 2**). At 3 DAP, the aborted seeds collapsed. Similar results were observed from the analysis of +/*rps9m-2* plants that half of seeds were aborted, and the endosperms arrested at two (17%) or four (21%) nuclei stage in the aborted seeds (**Table 2**). When we pollinated *+/rps9m-1* and wild type ovules with LIG1-GFP pollens, at 2 DAP, the paternal expression of LIG1-GFP was detected in both the embryo and endosperms in seeds produced from wild type and +/*rps9m-1* plants. But in +/*rps9m-1* siliques, nearly half of seeds were aborted at the same stage as we observed in +/*rps9m-1* self-pollination siliques (**Figures 7E–H** and **Table 2**). On the other hands, when +/*rps9m-1* pollens were used to pollinate wild type stigmas, embryo and endosperm developed normally (**Figures 7I,J** and **Table 2**). These results suggest that defects in female gametophyte lead to seed abortion in +/*rps9m-1* plants. When we pollinated WT and *+/rps9m-1* pistils with *pRPS9M-GUS* pollens. The paternally derived *pRPS9M-GUS* was expressed in the zygote and endosperms soon after fertilization in both wild type and *rps9m-1* ovules (**Figures 7K,L**), indicating the expression of the *RPS9M* allele in +/*rps9m-1* ovule is not silenced epigenetically.

**Table 2:** Percentage of aborted endosperm and embryo in *rps9m* mutant.

Female × male	Endosperm (%)				Embryo (%)			Unfertilized (%)	n
	1nuc	2nuc	4nuc	>4nuc	Zygote	2 cells	>2 cells		
+/*rps9m-1* × +/*rps9m-1*	7.4	19.5	14.6	49.7	21.9	19.6	49.7	8.8	437
+/*rps9m-2* × +/*rps9m-2*	2.7	17.2	21.4	51.3	14.5	26.8	51.3	7.4	452
+/*rps9m-1* × WT	4.8	21.7	18.4	48.6	21.2	23.7	48.6	6.5	463
+/*rps9m-2* × WT	3.1	16.8	24.5	50.7	15.3	29.1	50.7	4.9	446
WT × +/*rps9m-1*	0.0	0.0	1.6	92.7	1.6	0.0	92.7	5.7	375
WT × +/*rps9m-2*	0.0	0.0	1.9	92.9	0.0	1.9	92.9	5.2	381
WT × WT	0.0	0.0	0.0	94.6	0.0	0.0	94.6	5.4	249

*+, *RPS9M* allele; nuc, nucleus.*

## Discussion

In plants and animals, mutants of ribosomal protein genes display various specific developmental defects (Byrne, 2009; Gilbert, 2011; Horiguchi et al., 2011, 2012; Terzian and Box, 2013). Phenotypic diversity of ribosomal protein mutants may result from different patterns of gene expression or extraribosomal function of some ribosomal proteins (Ferreyra et al., 2010). Mitochondrion as a semi-autonomous organelle also contains ribosome to translate proteins encoded by mitochondrial genome. In *Arabidopsis*, several mutants in mitochondrial ribosomal protein genes have been reported (Portereiko et al., 2006b; Zhang et al., 2015). However, most of the mitochondrial ribosomal protein genes are still not functionally characterized. Here, we identified a mitochondrial ribosomal protein RPS9M that plays an important role in polar nuclei fusion, central cell development and endosperm proliferation. RPS9M physically interacts with ANK6, an ankyrin-repeat protein in mitochondria previously reported to be required for female gametophyte development and fertilization (Yu et al., 2010), suggesting that RPS9M may work together with ANK6 in controlling female gametophyte development.

Genetic characterization of heterozygous +/*rps9m* mutant plants indicates that *rps9m* mutation affects female gametophyte development. At the last development stage of *rps9m* female gametophyte, the two polar nuclei fail to fuse into one and the central cell vacuole is smaller than that in wild type (**Figure 5**). Lacking RPS9M probably leads to general dysfunction of mitochondria. Besides RPS9M, mutations in several other mitochondrial ribosomal proteins also caused gametophytic or seed development defects. These include HUELLENLOS (Skinner et al., 2001), RPS11 (NFD3) (Portereiko et al., 2006b), RPL21M (NFD1) (Portereiko et al., 2006b) and RPL18 (HEART STOPPER, HES) (Zhang et al., 2015). *HUELLENLOS* encode a mitochondrial ribosomal protein L14 that is homologous to eubacterial ribosome protein L14 and containing high similarity to cytosolic L23 and chloroplast ribosome L14 protein in *Arabidopsis*; mutation of *HUELLENLOS* lead to integuments reduced or absent, and ovule primordia collapse. Disruption of *RPS11* and *RPL21M* both cause polar nuclei fusion defection; moreover, *rpl21m* female gametophyte have a defect in fusion of sperm nuclei in both the egg cell and the central cell during fertilization. *HEART STOPPER* (*HES*) encode a mitochondrial ribosomal L18 protein, *hes* mutant showed no defections during gametophyte development but seed development was affected, embryos were arrested at the late globular stage and endosperms uncellularized in homozygous *hes* mutant seeds. Silencing of the nuclear *RPS10* gene disturbs the ratio between the small and large subunits of mitochondrial ribosome and changes the efficiency of translation of mRNAs for OXPHOS and ribosomal proteins (Kwasniak et al., 2013). Considering that RPS9M is a component of the mitoribosome, disruption of RPS9M may impact the integrity or stability of mitoribosome and thereby reducing the translation efficiency of some mitochondrial genes involved in mitochondrial function such as ATP synthesis, and ultimately affecting gametophyte development.

Although *RPS9M* is expressed in female gametophyte tissues throughout the developmental stages (**Figure 3**), disruption of *RPS9M* only impacts central cell development. No obvious morphological defects are observed in other cells such as egg cell and synergid cells of the *rps9m* female gametophyte (**Figure 5**). One plausible explanation for this phenomenon is that mutation of *RPS9M* impacts energy supply from mitochondria. The early development of *rps9m* female gametophyte is normal as the wild type mitochondria from the diploid MMC provide sufficient nutrients (ATPs, carbon skeletons) to the mutant female gametophyte. However, along with the further development of the *rps9m* female gametophyte, the new-born mitochondria have defect in energy supply. Inside the wild type embryo sac, the distribution of mitochondria is not uniform but largely concentrated in the central cell, whereas the egg cell and synergid cells contain only a few of mitochondria (Martin et al., 2014), suggesting that the central cell may need more energy for its development. The mutant central cell suffers more severely than other cells in the *rps9m* female gametophyte possibly due to defects in mitochondria failing to produce enough energy for polar nuclei fusion and central cell development.

Our data show that *rps9m* female gametophytes can be successfully fertilized (**Figure 6**), but the endosperm proliferation and embryo initiation were arrested after fertilization even when *rps9m* ovules were fertilized with wild type pollens (**Figure 7**), indicating that central cell development is critical for early endosperm development and embryo initiation. Recent studies provide evidence for the maternal control over embryogenesis and endosperm development in plant (Xin et al., 2012; Baroux and Grossniklaus, 2015). During central cell maturation, high levels of metabolic activities, large number of mitochondria, and enriched starch and lipid reserves are detected (Liu et al., 2010), suggesting that the central cell is primed for the immediate endosperm proliferation upon sperm cell delivery. But in *rps9m* ovules, the central cell maturation processes are defective and these may directly affect this “priming” process for endosperm development. Our data showed that the paternal-derived *de novo RPS9M* transcripts were detected in the zygote and endosperm cells immediately after fertilization in both *rps9m* ovules and wild type ovules, indicating that embryo initiation and endosperm development are independent of paternal source of RPS9M protein. Thus, the seed development defect in *rps9m* mutant may suffer from early defects in *rps9m* female gametes, which cannot be compensated by later supply of the RPS9M protein(s).

RPS9M physically interacts with ANK6 both *in vitro* and *in vivo* (**Figures 1, 2B**), suggesting that they are members of a protein complex in mitochondria during female gametophyte development. As an adaptor protein, ANK6 is presumed to mediate protein–protein interactions through its ANK repeat motifs, suggesting that more mitoribosomal proteins or other proteins may be recruited by ANK6 to work together with RPS9M during gametophyte developmental processes. In our previous work, an RNA polymerase transcription initiation factor, SIG5, was proven to physically interact with ANK6 in mitochondria, and the *sig5* ovules also show female gametophyte defections as *ank6*, indicating they are essential proteins in female gametophyte development (Yu et al., 2010). In eubacteria, the transcription of mRNAs and translation of protein processes are coupled, and some proteins and complexes including RNA polymerase subunits and cofactors may be involved in both transcription and protein synthesis (Kaczanowska and Ryden-Aulin, 2007). Future work will test the hypothesis that coupled transcription/translation processes in plant mitochondria may be operated by an complex, including SIG5 and RPS9M working together, to control the transcription and translation of some mitochondrial gene(s) during gametophyte development and fertilization.

## Accession Numbers

The accession numbers for the genes used for this article are as follows: *At3g49080* (*AtRPS9M*), *At5g61230* (*AtANK6*), and *At5g24120* (*AtSIG5*).

## Author Contributions

CL, FY, LC, SL, and DL designed the research. CL, FY, LT, XyH, HT, ZX, and XhH performed the research. CL, FY, LT, DL, SL, and LC analyzed data and wrote the paper.

## Conflict of Interest Statement

The authors declare that the research was conducted in the absence of any commercial or financial relationships that could be construed as a potential conflict of interest.

## Footnotes

1 www.sigmaaldrich.com

## References

[B1] Baroux C. Grossniklaus U. (2015). The maternal-to-zygotic transition in flowering plants: evidence, mechanisms, and plasticity. *Curr. Top. Dev. Biol.* 113 351–371. 10.1016/bs.ctdb.2015.06.005 26358878

[B2] Bemer M. Wolters Arts M. Grossniklaus U. Angenent G. C. (2008). The MADS domain protein DIANA acts together with AGAMOUS-LIKE80 to specify the central cell in *Arabidopsis* ovules. *Plant Cell* 20 2088–2101. 10.1105/tpc.108.058958 18713950 PMC2553608

[B3] Berger F. Chaudhury A. (2009). Parental memories shape seeds. *Trends Plant Sci.* 14 550–556. 10.1016/j.tplants.2009.08.003 19748816

[B4] Berger F. Grini P. E. Schnittger A. (2006). Endosperm: an integrator of seed growth and development. *Curr. Opin. Plant Biol.* 9 664–670. 10.1016/j.pbi.2006.09.015 17011228

[B5] Bonen L. Calixte S. (2006). Comparative analysis of bacterial-origin genes for plant mitochondrial ribosomal proteins. *Mol. Biol. Evol.* 23 701–712. 10.1093/molbev/msj080 16368778

[B6] Byrne M. E. (2009). A role for the ribosome in development. *Trends Plant Sci.* 14 512–519. 10.1016/j.tplants.2009.06.009 19716746

[B7] Chen Y. H. Li H. J. Shi D. Q. Yuan L. Liu J. Sreenivasan R. (2007). The central cell plays a critical role in pollen tube guidance in *Arabidopsis*. *Plant Cell* 19 3563–3577. 10.1105/tpc.107.053967 18055609 PMC2174880

[B8] Christensen C. A. Gorsich S. W. Brown R. H. Jones L. G. Brown J. Shaw J. M. (2002). Mitochondrial GFA2 is required for synergid cell death in Arabidopsis. *Plant Cell* 14 2215–2232. 10.1105/tpc.002170 12215516 PMC150766

[B9] Christensen C. A. King E. J. Jordan J. R. Drews G. N. (1997). Megagametogenesis in *Arabidopsis* wild type and the Gf mutant. *Sex. Plant Reprod.* 10 49–64. 10.1007/s004970050067

[B10] Clough S. J. Bent A. F. (1998). Floral dip: a simplified method for *Agrobacterium*-mediated transformation of *Arabidopsis thaliana*. *Plant J.* 16 735–743. 10.1046/j.1365-313x.1998.00343.x 10069079

[B11] Feng Z. Zhang B. Ding W. Liu X. Yang D. L. Wei P. (2013). Efficient genome editing in plants using a CRISPR/Cas system. *Cell Res.* 23 1229–1232. 10.1038/cr.2013.114 23958582 PMC3790235

[B12] Ferreyra M. L. F. Pezza A. Biarc J. Burlingame A. L. Casati P. (2010). Plant L10 ribosomal proteins have different roles during development and translation under ultraviolet-B stress. *Plant Physiol.* 153 1878–1894. 10.1104/pp.110.157057 20516338 PMC2923885

[B13] Gilbert W. V. (2011). Functional specialization of ribosomes? *Trends Biochem. Sci.* 36 127–132. 10.1016/j.tibs.2010.12.002 21242088 PMC3056915

[B14] Horiguchi G. Molla Morales A. Perez J. M. Kojima K. Robles P. Ponce M. R. (2011). Differential contributions of ribosomal protein genes to *Arabidopsis thaliana* leaf development. *Plant J.* 65 724–736. 10.1111/j.1365-313X.2010.04457.x 21251100

[B15] Horiguchi G. Van Lijsebettens M. Candela H. Micol J. L. Tsukaya H. (2012). Ribosomes and translation in plant developmental control. *Plant Sci.* 191 24–34. 10.1016/j.plantsci.2012.04.008 22682562

[B16] Huh J. H. Bauer M. J. Hsieh T. F. Fischer R. L. (2008). Cellular programming of plant gene imprinting. *Cell* 132 735–744. 10.1016/j.cell.2008.02.018 18329361

[B17] Ingouff M. Hamamura Y. GourgueS M. Higashiyama T. Berger F. (2007). Distinct dynamics of HISTONE3 variants between the two fertilization products in plants. *Curr. Biol.* 17 1032–1037. 10.1016/j.cub.2007.05.019 17555967

[B18] Ingouff M. Sakata T. Li J. Sprunck S. Dresselhaus T. Berger F. (2009). The two male gametes share equal ability to fertilize the egg cell in *Arabidopsis thaliana*. *Curr. Biol.* 19 R19–R20. 10.1016/j.cub.2008.11.025 19138583

[B19] Jefferson R. A. Kavanagh T. A. Bevan M. W. (1987). GUS fusions: beta-glucuronidase as a sensitive and versatile gene fusion marker in higher plants. *EMBO J.* 6 3901–3907. 3327686 10.1002/j.1460-2075.1987.tb02730.x PMC553867

[B20] Jiang W. Zhou H. Bi H. Fromm M. Yang B. Weeks D. P. (2013). Demonstration of CRISPR/Cas9/sgRNA-mediated targeted gene modification in Arabidopsis, tobacco, sorghum and rice. *Nucleic Acids Res.* 41:e188. 10.1093/nar/gkt780 23999092 PMC3814374

[B21] Kaczanowska M. Ryden-Aulin M. (2007). Ribosome biogenesis and the translation process in *Escherichia coli*. *Microbiol. Mol. Biol. Rev.* 71 477–494. 10.1128/MMBR.00013-07 17804668 PMC2168646

[B22] Kaegi C. Baumann N. Nielsen N. Stierhof Y. D. Gross-Hardt R. (2010). The gametic central cell of *Arabidopsis* determines the lifespan of adjacent accessory cells. *Proc. Natl. Acad. Sci. U.S.A.* 107 22350–22355. 10.1073/pnas.1012795108 21135240 PMC3009827

[B23] Kaegi C. Gross-Hardt R. (2007). How females become complex: cell differentiation in the gametophyte. *Curr. Opin. Plant Biol.* 10 633–638. 10.1016/j.pbi.2007.07.011 17851110

[B24] Kwasniak M. Majewski P. Skibior R. Adamowicz A. Czarna M. Sliwinska E. (2013). Silencing of the nuclear *RPS10* gene encoding mitochondrial ribosomal protein alters translation in *Arabidopsis* mitochondria. *Plant Cell* 25 1855–1867. 10.1105/tpc.113.111294 23723321 PMC3694710

[B25] Liu J. Qu L. J. (2008). Meiotic and mitotic cell cycle mutants involved in gametophyte development in *Arabidopsis*. *Mol. Plant* 1 564–574. 10.1093/mp/ssn033 19825562

[B26] Liu Y. Yan Z. Q. Chen N. Di X. T. Huang J. J. Guo G. Q. (2010). Development and function of central cell in angiosperm female gametophyte. *Genesis* 48 466–478. 10.1002/dvg.20647 20506265

[B27] Ma H. Sundaresan V. (2010). Development of flowering plant gametophytes. *Plant Dev.* 91 379–412. 10.1016/S0070-2153(10)91013-2 20705189

[B28] Mao Y. Zhang H. Xu N. Zhang B. Gou F. Zhu J. K. (2013). Application of the CRISPR-Cas system for efficient genome engineering in plants. *Mol. Plant* 6 2008–2011. 10.1093/mp/sst121 23963532 PMC3916745

[B29] Martin M. V. Distefano A. M. Bellido A. Cordoba J. P. Soto D. Pagnussat G. C. (2014). Role of mitochondria during female gametophyte development and fertilization in *A. thaliana*. *Mitochondrion* 19 350–356. 10.1016/j.mito.2014.01.005 24512842

[B30] Martin M. V. Fiol D. F. Sundaresan V. Zabaleta E. J. Pagnussat G. C. (2013). *oiwa*, a female gametophytic mutant impaired in a mitochondrial manganese-superoxide dismutase, reveals crucial roles for reactive oxygen species during embryo sac development and fertilization in *Arabidopsis*. *Plant Cell* 25 1573–1591. 10.1105/tpc.113.109306 23653473 PMC3694693

[B31] Maruyama D. Endo T. Nishikawa S. I. (2010). BiP-mediated polar nuclei fusion is essential for the regulation of endosperm nuclei proliferation in *Arabidopsis thaliana*. *Proc. Natl. Acad. Sci. U.S.A.* 107 1684–1689. 10.1073/pnas.0905795107 20080634 PMC2824368

[B32] North H. Baud S. Debeaujon I. Dubos C. Dubreucq B. Grappin P. (2010). Arabidopsis seed secrets unravelled after a decade of genetic and omics-driven research. *Plant J.* 61 971–981. 10.1111/j.1365-313X.2009.04095.x 20409271

[B33] Portereiko M. F. Lloyd A. Steffen J. G. Punwani J. A. Otsuga D. Drews G. N. (2006a). *AGL80* is required for central cell and endosperm development in *Arabidopsis*. *Plant Cell* 18 1862–1872. 16798889 10.1105/tpc.106.040824 PMC1533969

[B34] Portereiko M. F. Sandaklie-Nikolova L. Lloyd A. Dever C. A. Otsuga D. Drews G. N. (2006b). *NUCLEAR FUSION DEFECTIVE1* encodes the Arabidopsis RPL21M protein and is required for karyogamy during female gametophyte development and fertilization. *Plant Physiol.* 141 957–965. 16698901 10.1104/pp.106.079319 PMC1489897

[B35] Sheen J. (2001). Signal transduction in maize and Arabidopsis mesophyll protoplasts. *Plant Physiol.* 127 1466–1475. 10.1104/pp.010820 11743090 PMC1540179

[B36] Skinner D. J. Baker S. C. Meister R. J. Broadhvest J. Schneitz K. Gasser C. S. (2001). The Arabidopsis *HUELLENLOS* gene, which is essential for normal ovule development, encodes a mitochondrial ribosomal protein. *Plant Cell* 13 2719–2730. 10.1105/tpc.13.12.2719 11752383 PMC139484

[B37] Steffen J. G. Kang I. H. Portereiko M. F. Lloyd A. Drews G. N. (2008). AGL61 interacts with AGL80 and is required for central cell development in Arabidopsis. *Plant Physiol.* 148 259–268. 10.1104/pp.108.119404 18599653 PMC2528130

[B38] Sundaresan V. Alandete-Saez M. (2010). Pattern formation in miniature: the female gametophyte of flowering plants. *Development* 137 179–189. 10.1242/dev.030346 20040485

[B39] Terzian T. Box N. (2013). Genetics of ribosomal proteins: ”curiouser and curiouser”. *PLOS Genet.* 9:e1003300. 10.1371/journal.pgen.1003300 23382707 PMC3561088

[B40] Twell D. Wing R. Yamaguchi J. McCormick S. (1989). Isolation and expression of an anther-specific gene from tomato. *Mol. Gen. Genet.* 217 240–245. 10.1007/BF02464887 2770694

[B41] Wu J. J. Peng X. B. Li W. W. He R. Xin H. P. Sun M. X. (2012). Mitochondrial GCD1 dysfunction reveals reciprocal cell-to-cell signaling during the maturation of *Arabidopsis* female gametes. *Dev. Cell* 23 1043–1058. 10.1016/j.devcel.2012.09.011 23085019

[B42] Xin H. P. Zhao J. Sun M. X. (2012). The maternal-to-zygotic transition in higher plants. *J. Integr. Plant Biol.* 54 610–615. 10.1111/j.1744-7909.2012.01138.x 22731521

[B43] Yadegari R. Drews G. N. (2004). Female gametophyte development. *Plant Cell* 16(Suppl.) S133–S141. 10.1105/tpc.018192 15075395 PMC2643389

[B44] Yang W. C. Shi D. Q. Chen Y. H. (2010). Female gametophyte development in flowering plants. *Annu. Rev. Plant Biol.* 61 89–108. 10.1146/annurev-arplant-042809-112203 20192738

[B45] Yu F. Shi J. Zhou J. Gu J. Chen Q. Li J. (2010). ANK6, a mitochondrial ankyrin repeat protein, is required for male-female gamete recognition in *Arabidopsis thaliana*. *Proc. Natl. Acad. Sci. U.S.A.* 107 22332–22337. 10.1073/pnas.1015911107 21123745 PMC3009778

[B46] Zhang H. Luo M. Day R. C. Talbot M. J. Ivanova A. Ashton A. R. (2015). Developmentally regulated *HEART STOPPER*, a mitochondrially targeted L18 ribosomal protein gene, is required for cell division, differentiation, and seed development in *Arabidopsis*. *J. Exp. Bot.* 66 5867–5880. 10.1093/jxb/erv296 26105995 PMC4566979

[B47] Zhang H. Zhang J. Wei P. Zhang B. Gou F. Feng Z. (2014). The CRISPR/Cas9 system produces specific and homozygous targeted gene editing in rice in one generation. *Plant Biotechnol. J.* 12 797–807. 10.1111/pbi.12200 24854982

