# Supplementary material for: RPS9M, a Mitochondrial Ribosomal Protein, Is Essential for Central Cell Maturation and Endosperm Development in Arabidopsis
Source: Front Plant Sci. 2017 Dec 22;8:2171. doi: 10.3389/fpls.2017.02171 (PMC5744018; doi:10.3389/fpls.2017.02171)
Supplement: Supplementary file 1 [file Table_1.DOCX]

**Table S1.** Segregation of *RPS9M/rps9m* alleles by selfed-cross

| Lines | | Progeny Genotypes | | | Expected Ratio | Observed Ratio |
| --- | --- | --- | --- | --- | --- | --- |
|  |  | +/+ | +/- | -/- |  |  |
| +/*rps9m-1* | 1＃ | 102 | 21 | 0 | 1:2:1 | 1:0.21:0^a^ |
|  | 2＃ | 91 | 17 | 0 | 1:2:1 | 1:0.19:0^a^ |
|  | 3＃ | 134 | 30 | 0 | 1:2:1 | 1:0.22:0^a^ |
|  | 4＃ | 119 | 24 | 0 | 1:2:1 | 1:0.20:0^a^ |
|  | 5＃ | 92 | 20 | 0 | 1:2:1 | 1:0.22:0^a^ |
| +/*rps9m-2* | 1＃ | 115 | 34 | 0 | 1:2:1 | 1:0.30:1^a^ |
|  | 2＃ | 127 | 35 | 0 | 1:2:1 | 1:0.28:0^a^ |
|  | 3＃ | 108 | 28 | 0 | 1:2:1 | 1:0.26:0^a^ |
|  | 4＃ | 113 | 29 | 0 | 1:2:1 | 1:0.26:0^a^ |
|  | 5＃ | 97 | 24 | 0 | 1:2:1 | 1:0.25:0^a^ |

^a^ Significantly different from the expected 1:2:1 segregation ratio (P < 0.001);

+, *RPS9M* allele; -, *rps9m* allele.
