# Supplementary material for: RPS9M, a Mitochondrial Ribosomal Protein, Is Essential for Central Cell Maturation and Endosperm Development in Arabidopsis
Source: Front Plant Sci. 2017 Dec 22;8:2171. doi: 10.3389/fpls.2017.02171 (PMC5744018; doi:10.3389/fpls.2017.02171)
Supplement: Supplementary file 2 [file Table_2.DOCX]

**Table S2.** Primers used in this study

| Primers | Sequence (5’-3’) | Purpose |
| --- | --- | --- |
| RPS9M-F | GTTCTTCTTCGAACTTCTGCA | For RPS9M cDNA cloning |
| RPS9M-R | GGACATTCCTTAACATTTTGAT |  |
| RPS9M-PF | GCGTCGACGAATCGTATTGTATCGCCC | For GUS expression vector construct |
| RPS9M-PR | CGGGATCCGATGGTGAAGACGAACACTTG |  |
| RPS9M-GF | GCTCTAGAATGCTCTCTCGTTTATTCCTTA | For GFP expression vector construct |
| RPS9M-GR | CGGATCCACGTTTGACCCATTGGAAG |  |
| RPS9M-BIFCF | CGGAATTCATGCTCTCTCGTTTATTCCTTA | For BIFC vector construct |
| RPS9M-BIFCR | CGGGATCCAACGTTTGACCCATTGGA |  |
| ANK6-BiFCF | CGGAATTCATGCTCCAAGAACCGTC |  |
| ANK6-BiFCR | CGGGATCCGTTGTCAGAACTGGAAGATGT |  |
| RPS9M-sg1F | GATTGGTGGAGAGGGGAAATGGC | For CRISPR/Cas9 plant expression vector construct |
| RPS9M-sg1R | AAACGCCATTTCCCCTCTCCACC |  |
| RPS9M-sg2F | GATTGGATTGAGACTAGTCGCGAG |  |
| RPS9M-sg2R | AAACCTCGCGACTAGTCTCAATCC |  |
| RPS9M-TF | ATTTGAGGCGATTGGATTCTATG | For *rps9m* mutant identification |
| RPS9M-TR | CTATTCTCAAAGCTAAACGGCACT |  |
| ANK6-Y2HF | CCCATATGATGCTCCAAGAACCGTC | For yeast two-hybrid analysis victor construction |
| ANK6-Y2HR | CGGAATTCGTTGTCAGAACTGGAAGATGT |  |
| RPS9M-1-Y2HF | GGAATTCTCATCGAAATCAAATTCTCAGA |  |
| RPS9M-1-Y2HR | CGGATCCGACGTTTGACCCATTGGAAG |  |
| RPS9M-2-Y2HF | GGAATTCTCATCGAAATCAAATTCTCAGA |  |
| RPS9M-2-Y2HR | CGGATCCGAAGGTCTCCAAATGCTCGAT |  |
| RPS9M-3-Y2HF | GGAATTCGGTCCTGATCGAGCATTTG |  |
| RPS9M-3-Y2HR | CGGATCCGACGTTTGACCCATTGGAAG |  |
