# Supplementary material for: RPS9M, a Mitochondrial Ribosomal Protein, Is Essential for Central Cell Maturation and Endosperm Development in Arabidopsis
Source: Front Plant Sci. 2017 Dec 22;8:2171. doi: 10.3389/fpls.2017.02171 (PMC5744018; doi:10.3389/fpls.2017.02171)
Supplement: Supplementary file 3 [file Table_3.DOCX]

**Table S3.** Central vacuole size of wild type, *rps9m-1* and *rps9m-2* ovules.

| Stage | Vacuole Size (μm^2^)^a^ | | |
| --- | --- | --- | --- |
|  | WT | *rps9m-1* | *rps9m-2* |
| Early FG5 | 343±40.7 | 302±46.3 | 297±49.5 |
| Later FG5 | 457±31.4 | 349±42.9^b^ | 335±40.6^b^ |
| FG6 | 381±39.7 | 381±39.7^b^ | 352±43.2^b^ |
| FG7 | 510±28.5 | 390±32.7^b^ | 365±38.9^b^ |

^a^ The data represented the mean size of vacuole from 20 ovules.

^b^ Significantly difference based on Student's *t*-test, P < 0.05.
