# Supplementary material for: RPS9M, a Mitochondrial Ribosomal Protein, Is Essential for Central Cell Maturation and Endosperm Development in Arabidopsis
Source: Front Plant Sci. 2017 Dec 22;8:2171. doi: 10.3389/fpls.2017.02171 (PMC5744018; doi:10.3389/fpls.2017.02171)
Supplement: Supplementary file 4 [file Table_4.DOCX]

**Table S4.** Synergid vacuole size of wild type, *rps9m-1* and *rps9m-2* ovules.

|  | Synergid Vacuole Size (μm^2^)^a^ |
| --- | --- |
| WT | 103±10.7 |
| *rps9m-1* | 92±16.2 |
| *rps9m-2* | 96±14.6 |

^a^ The data represented the mean size of vacuole from 20 ovules.
