# Supplementary material for: RPS9M, a Mitochondrial Ribosomal Protein, Is Essential for Central Cell Maturation and Endosperm Development in Arabidopsis
Source: Front Plant Sci. 2017 Dec 22;8:2171. doi: 10.3389/fpls.2017.02171 (PMC5744018; doi:10.3389/fpls.2017.02171)
Supplement: Supplementary file 5 [file Image_1.PDF]

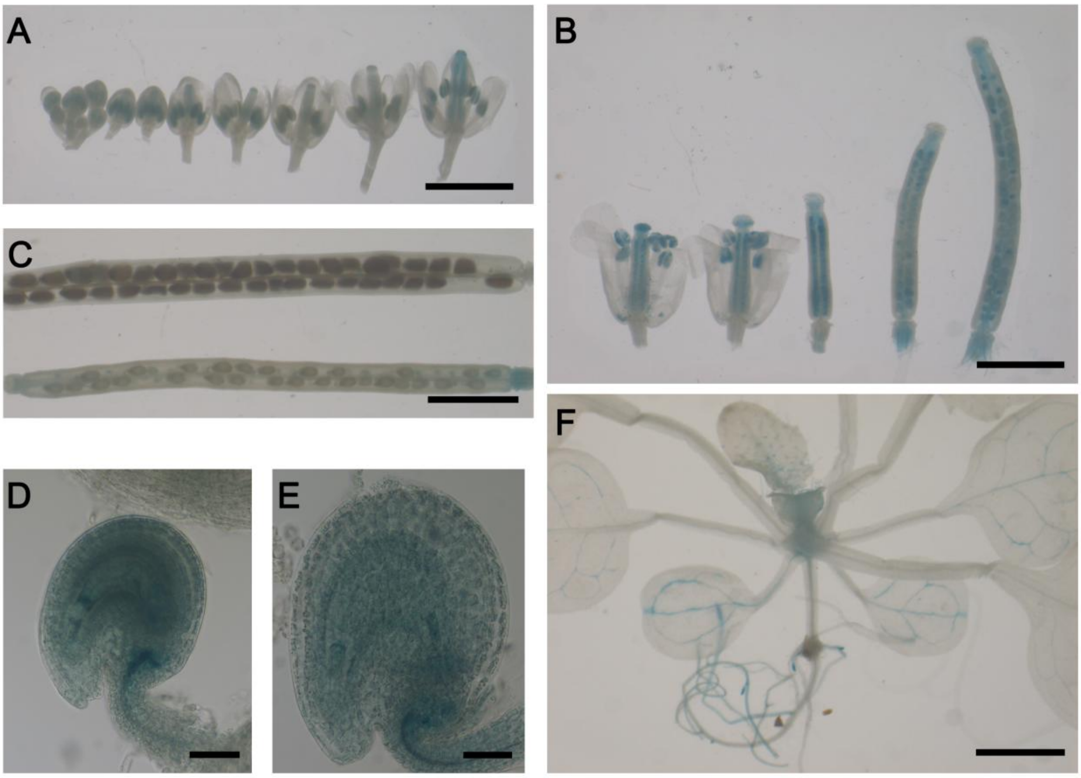

Figure S1. Expression patterns of RPS9M by GUS reporter analysis. Histochemical analysis of *AtRPS9M* promoter-driven GUS reporter expression in transgenic *Arabidopsis* plants. (A-B) young siliques and flowers at different development stages. (C) mature siliques. (D) ovule at 1 DAP. (E) seed at 2 DAP. (F) 3-week-old plants. Bars = 2 mm in A-D and F; 20 μm in D-E.
