# Supplementary material for: RPS9M, a Mitochondrial Ribosomal Protein, Is Essential for Central Cell Maturation and Endosperm Development in Arabidopsis
Source: Front Plant Sci. 2017 Dec 22;8:2171. doi: 10.3389/fpls.2017.02171 (PMC5744018; doi:10.3389/fpls.2017.02171)
Supplement: Supplementary file 6 [file Image_2.PDF]

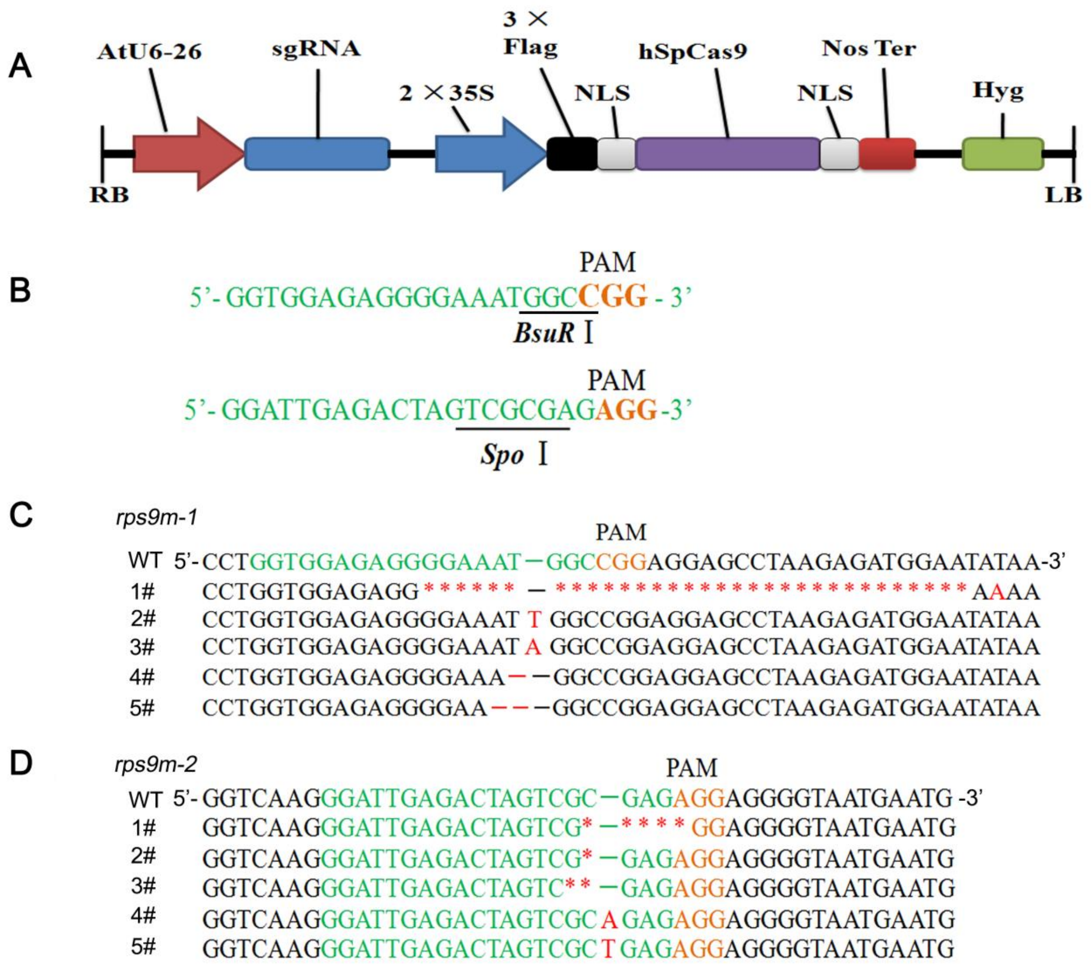

Figure S2. Creation and identification of the *rps9m* mutation. The *rps9m* mutation was created by using CRISPR-Cas9 editing system. (A) Schematic diagram of the sgRNA and hSpCas9 expression cassettes in a single binary vector for *Agrobacterium*-mediated stable transformation of *Arabidopsis*. (B) The sequences of the two target sites from the *AtRPS9M* locus. The PAM and the restriction enzyme site (underlined) are indicated. (C) Alignment of sequences of the *rps9m-1* alleles identified by sequencing. The mutated alleles include deletions and insertions. (D) Alignment of sequences of the *rps9m-2* alleles identified by sequencing.
