# Supplementary material for: RPS9M, a Mitochondrial Ribosomal Protein, Is Essential for Central Cell Maturation and Endosperm Development in Arabidopsis
Source: Front Plant Sci. 2017 Dec 22;8:2171. doi: 10.3389/fpls.2017.02171 (PMC5744018; doi:10.3389/fpls.2017.02171)
Supplement: Supplementary file 7 [file Image_3.PDF]

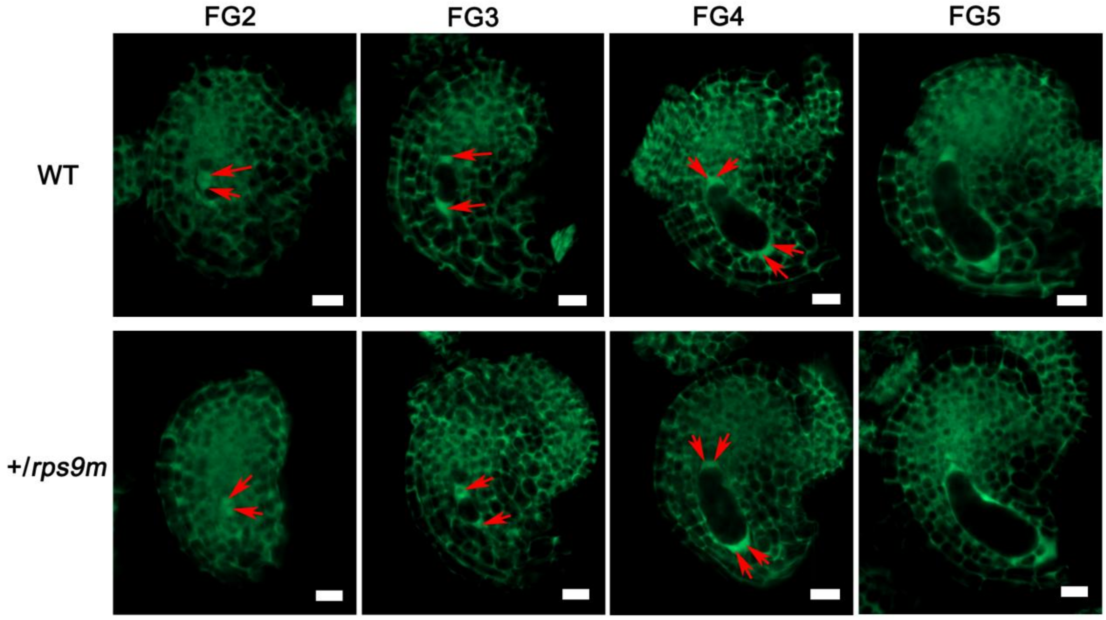

Figure S3. Arabidopsis female gametophyte development in wild-type and +/*rps9m*. CLSM analysis of ovules at FG2, FG3, FG4 and FG5 from WT and +/*rps9m*. Bars = 10 μm.
