# Supplementary material for: RPS9M, a Mitochondrial Ribosomal Protein, Is Essential for Central Cell Maturation and Endosperm Development in Arabidopsis
Source: Front Plant Sci. 2017 Dec 22;8:2171. doi: 10.3389/fpls.2017.02171 (PMC5744018; doi:10.3389/fpls.2017.02171)
Supplement: Supplementary file 8 [file Image_4.PDF]

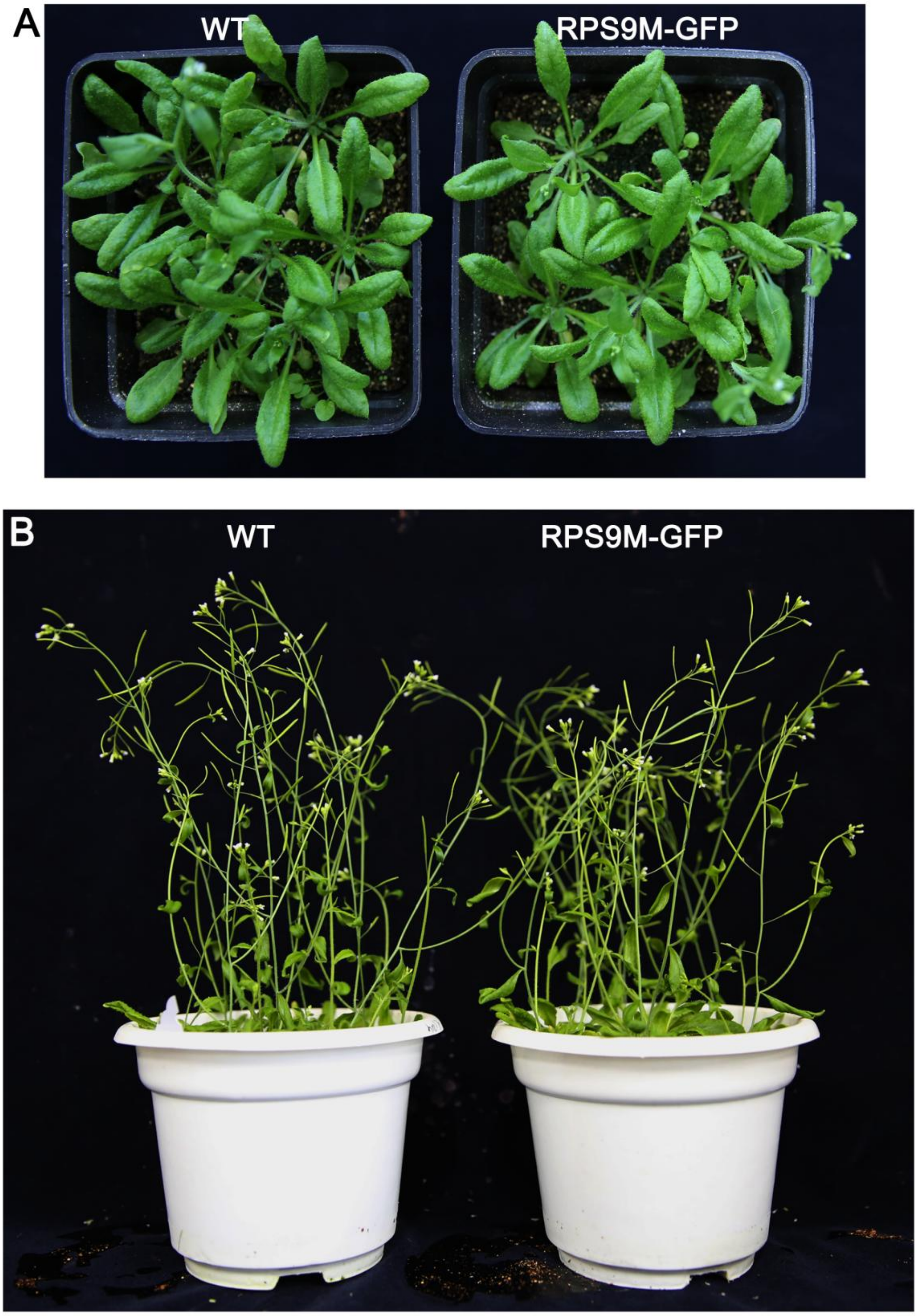

Figure S4. Phenotype of wild type and RPS9M-GFP plants. (A) Vegetative growth phenotype of wild type and 35S-*RPS9M-GFP* plants. (B) Reproductive growth phenotype of wild type and 35S-*RPS9M-GFP* plants.
